# Immunoinformatics Approach for Epitope-Based Vaccine Design: Key Steps for Breast Cancer Vaccine

**DOI:** 10.3390/diagnostics12122981

**Published:** 2022-11-28

**Authors:** Aisyah Fitriannisa Prawiningrum, Rafika Indah Paramita, Sonar Soni Panigoro

**Affiliations:** 1 Master’s Programme in Biomedical Sciences, Faculty of Medicine, Universitas Indonesia, Jakarta 10430, Indonesia; 2 Bioinformatics Core Facilities—IMERI, Faculty of Medicine, Universitas Indonesia, Jakarta 10430, Indonesia; 3 Doctoral Program in Biomedical Sciences, Faculty of Medicine, Universitas Indonesia, Jakarta 10430, Indonesia; 4 Department of Medical Chemistry, Faculty of Medicine, Universitas Indonesia, Jakarta 10430, Indonesia; 5 Surgical Oncology Division, Department of Surgery, Faculty of Medicine, Universitas Indonesia, Jakarta 10430, Indonesia

**Keywords:** breast cancer, immunoinformatics, vaccine, epitope

## Abstract

Vaccines are an upcoming medical intervention for breast cancer. By targeting the tumor antigen, cancer vaccines can be designed to train the immune system to recognize tumor cells. Therefore, along with technological advances, the vaccine design process is now starting to be carried out with more rational methods such as designing epitope-based peptide vaccines using immunoinformatics methods. Immunoinformatics methods can assist vaccine design in terms of antigenicity and safety. Common protocols used to design epitope-based peptide vaccines include tumor antigen identification, protein structure analysis, T cell epitope prediction, epitope characterization, and evaluation of protein–epitope interactions. Tumor antigen can be divided into two types: tumor associated antigen and tumor specific antigen. We will discuss the identification of tumor antigens using high-throughput technologies. Protein structure analysis comprises the physiochemical, hydrochemical, and antigenicity of the protein. T cell epitope prediction models are widely available with various prediction parameters as well as filtering tools for the prediction results. Epitope characterization such as allergenicity and toxicity can be done in silico as well using allergenicity and toxicity predictors. Evaluation of protein–epitope interactions can also be carried out in silico with molecular simulation. We will also discuss current and future developments of breast cancer vaccines using an immunoinformatics approach. Finally, although prediction models have high accuracy, the opposite can happen after being tested in vitro and in vivo. Therefore, further studies are needed to ensure the effectiveness of the vaccine to be developed. Although epitope-based peptide vaccines have the disadvantage of low immunogenicity, the addition of adjuvants can be a solution.

## 1. Introduction

According to the Global Cancer Observatory in 2020, around 11.7% of new worldwide cases of cancer are breast cancer. Approximately 6.9% of deaths by cancer were caused by breast cancer in 2020 all around the world [1]. In Indonesia, 19.2% of cancer cases are breast cancer, making it the most prevalent cancer [2]. Breast cancer has been recorded as the type of cancer that causes the highest mortality in women due to its high incidence. Given the devastating implications of the disease and the growing number of cases, many scientists and research organizations have dedicated their efforts to the fight against breast cancer [3]. Several suggestions, such as living a healthy lifestyle, getting regular exercise, managing body weight, and quitting smoking, should be taken into consideration as preventative measures. Many healthcare organizations advise annual and routine mammography after the age of 40 for early disease identification. As a result, the sickness would be diagnosed early, and the therapy would begin before it spread to other bodily areas. There are currently three ways to treat breast cancer: surgical ablation, radiotherapy, and chemotherapy. Each of these treatments can have bad side effects or even cause the cancer to reoccur [4]. Many laboratories are working on breast cancer vaccines to generate a long-lasting anticancer response with few side effects.

Contrary to the traditional idea of immunizing against infectious diseases only, the concept of vaccines can be adopted for both cancer prevention and therapy. A cancer vaccine can theoretically treat the malignancy by inducing T cell anti-tumor mechanisms. It causes minimal effects compared to conventional cancer therapy. Radiotherapy, chemotherapy, and endocrine therapies can cause adverse effects such as skin toxicity, peripheral neuropathy, hair loss, infertility, impaired cognitive function, and tiredness [5]. However, creating a cancer vaccine has proven to be difficult, partly because there are so many possible antigens that the immune system could attack. Many of these antigens may also develop before, during, or after the neoplastic process. Despite optimistic advancements in cancer immunotherapy, the search for vaccine target identification techniques continues to this day.

Though there is no widely accepted universal technique or instrument for rationally creating vaccines, researchers agree on various steps needed during the design process. Computational methods can be used to significantly reduce the time and cost of developing vaccines by mapping thousands of biological components in silico. Recent studies have highlighted the influence of these techniques on vaccine design from a variety of perspectives, including proteome retrieval, epitope prediction, epitope selection, molecular interaction, and immune response simulation [6,7,8]. This set of techniques to select potential vaccine targets and simulate immune responses is often referred to as immunoinformatics.

The immunoinformatics approach to vaccine design relies heavily on antigen identification and the selection of epitopes that can induce an immune response. With various optimized algorithms and high-throughput genomics analysis, antigen search, molecular docking, and model simulations to predict immune responses can be carried out more quickly. This will of course reduce the intensity of testing work in the laboratory [9]. In this paper, we will discuss further the immunoinformatics approach that can be applied as a preliminary study in designing vaccines for breast cancer.

## 2. Design Strategy for Breast Cancer Vaccine

Cancer vaccines are now focusing on subunit components rather than cell-based or virus-based vaccines [10]. The immunogenicity of peptide-based vaccinations is low due to the limitations of HLA polymorphism and the tiny size of antigen epitopes themselves. It is frequently difficult to elicit a strong immunological response, which leads to immune tolerance. Adjuvants are used in conjunction with peptide-based vaccinations to improve the overall immune response. Not all protein antigen sites are similarly immunogenic to B and T cells. Instead of inactivated tumor cells, peptide-based vaccines target key neutralizing epitopes to get a more targeted immune response [11]. Cancer vaccines based on peptides often require both CD8+ T cell epitopes and CD4+ T cell epitopes. CD8+ T cell epitopes activate CTLs’ tumor immunity via the antigen cross-presentation pathway, whereas CD4+ T cells stimulate helper T cells to keep CTLs functional [12].

The length of the peptide chain has a significant impact on the performance of the peptide vaccination. CD8+ T cell epitopes are typically short peptides with a short half-life in vivo. This peptide is directly applied to the HLA-I molecules of APCs or other nucleus cells, removing the need for processing in specialized APCs. CTL activation is limited by the lack of costimulatory molecules, which are needed for CD8+ T cells to work well [13]. As a result, short peptides frequently activate CTLs and even induce CTL tolerance [14]. Furthermore, shorter peptides are often constrained by HLA types. Long peptides, as opposed to short peptides, enable greater coverage of HLA, encompassing many epitopes while also supporting motif recognition and binding to increase immunogenicity. Long peptides must be processed by APCs before being loaded directly onto HLA molecules [15]. A portion of the lengthy peptides is digested by the endosomal route after internalization, loaded onto HLA-II molecules, and identified by CD4+ T helper cells. The remaining portions enter the cytoplasmic or vacuolar route and are presented to CD8+ T lymphocytes via HLA-I molecules [16]. Long peptide vaccines have a greater chance of eliciting long-lasting and effective anti-tumor activity responses. Short peptides are often created via chemical synthesis, but lengthy peptides are generally created using protein expression systems. Immunogenicity differs among recombinant protein subunit vaccines depending on the expression platform. Cancer vaccines have been produced using a variety of expression platforms, including *Escherichia coli* (*E. coli*) [17], plants [18], yeasts [19], insect cells [20], and mammalian cells [21]. Mammalian cell proteins are the most similar to natural tumor antigens.

This review will focus on two types of antigens, tumor-associated antigens and tumor-specific antigens. Both types of antigens can be targeted in breast cancer vaccine design with several limitations.

(a) Tumor-Associated Antigens (TAA)

Tumor-associated antigens are molecules derived from unmutated proteins and are recognized by TCRs. They are associated with tumor cells because tumor cells produce them at significantly high levels. TAAs are useful for producing a single vaccination that can be made in huge quantities and disseminated to many patients as a one-for-all strategy. One of the most difficult issues is ensuring that TAAs elicit the optimal immune response. The immune system is meticulously calibrated to ensure that it does not harm the body. When this calibration fails, autoimmune disorders develop. Some TAAs may be detected in healthy tissues, but at low levels. As a result, tumor-associated antigens may not elicit an immunological response because the immune system regards them as foreign. On the other hand, TAAs may evade human immune tolerance systems. This could cause immune cells to target other sections of the body, potentially resulting in toxicity and safety concerns [22]. Currently, there are several peptide vaccines for breast cancer that are being developed based on TAAs such as E75 [23], GP2 [24], and AE37 [25].

(b) Tumor-Specific Antigens (TSA)

Tumor-specific antigens, also known as neoantigens, are a repertoire of peptides presented on tumor cells that may be selectively recognized by neoantigen-specific T cell receptors (TCRs) in the context of human leukocyte antigen (HLA) molecules [26,27,28]. Tumor neoantigen is an aberrant protein that is completely missing from normal human organs/tissues. Tumor neoantigens can arise from a range of nonsynonymous genetic modifications, including single-nucleotide variations (SNVs), insertions and deletions (indel), gene fusions, frameshift mutations, and structural variants (SVs) [26]. The main constraint of cancer vaccines based on altered neoantigens is that they are strictly personalized, and their discovery necessitates a combination of high-throughput genomics, proteomics, and immunomics screening technologies that are presently not applicable on a broad scale. Furthermore, the success of such a highly customized strategy may be hampered by tumors’ rapid mutational rate, which leads to the continual creation of new target mutated neoantigens and, as a result, cancer immune evasion.

## 3. Immune Response to Epitope-Based Peptide Vaccine

Not all parts of the antigen can be recognized by the immune system. The fraction of antigens that can interact with B cell and T cell receptors as well as free antibody molecules are called epitopes or antigenic determinants. The size of an epitope is in the range of 5–15 amino acids [29,30]. One protein usually has many epitopes with different specificities. This is because the protein structure generally has a long peptide chain and undergoes folding due to interactions between residues in it. It is this protein complex that makes proteins more immunogenic than polysaccharides [31].

To induce a response from T cells, epitope-based peptide vaccines must be able to bind to the T cell receptor (TCR) and be presented by antigen-presenting cells (APC) via human leukocyte antigen (HLA) classes I and II. HLA is a surface molecule that functions to present antigens that have undergone proteasomal degradation in cells and become short peptides (8–11 residues for HLA class I and 11–30 for HLA class II) [32]. HLA class I is expressed by all nucleated cells and interacts with the CD8 protein of CD8+ T cells. Meanwhile, HLA class II is only expressed by APC and only interacts with CD4 from CD4+ T cells. HLA is encoded by genes that are highly polymorphic, meaning that many different alleles can be found in a population. This can cause different adaptive immune reactions in different individuals. Chowell et al. [33] studied over 1500 patients and discovered that heterozygosity at the HLA-I loci was related to greater survival than homozygosity for one or more HLA-I genes. As a result, certain HLA-I mutations may affect immune recognition as well as the development of epitope-based cancer vaccines and immunotherapies. 

To observe the polymorphism of HLA, the IEDB population coverage is often used to calculate population coverage of epitopes [34] based on HLA allele (genotypic) frequencies obtained from the dbMHC database (http://www.ncbi.nlm.nih.gov/mhc/, accessed on 30 October 2022). The Population Coverage Calculation program allows custom populations with allele frequencies defined by users in addition to the allele frequencies acquired from the dbMHC database. Multiple population coverages can be estimated at the same time, yielding an average population coverage. Because HLA class I- and HLA class II-restricted T cell epitopes elicit immune responses from two distinct T cell populations (CTL and HTL, respectively), the program offers three calculation modes to accommodate different coverage modes: (1) class I separately, (2) class II separately, and (3) class I and class II combined. A histogram is created for each population coverage to summarize the percentage distribution of people as a function of the number of epitope/HLA combinations detected. Another database to use is the Allele Frequency Net Database (AFND, http://www.allelefrequencies.net, accessed on 30 October 2022), which provides information on the frequency of immune-related genes and their matching alleles from over 1700 population samples from throughout the world, totaling over 10 million unrelated people [35]. 

The AFNDB and IEDB population coverage tools are regularly utilized as reference sources of HLA frequencies by the scientific community worldwide [36]; therefore, keeping them up to date is critical. However, they rely on the scientific community users for data gathering and curation [36,37]. For some continents, these databases generally contain tiny datasets from ethnic groups that are not typical of the country’s variety, resulting in an erroneous distribution of HLA frequencies at the moment. A literature review by Requena et al. [38] provides revised HLA allele frequencies for South America, correcting previously misrepresented alleles. Another study also updated HLA allele frequencies for South Africa by auditing their HLA-typing files for the period 2005–2019 [39]. The frequent updates on HLA allele frequencies for each continent and country will have a great impact on vaccine design.

The antigen recognition process begins with a specific epitope-specific TCR binding to the peptide–HLA complex (pHLA). This recognition process is also modulated by interactions with other surface proteins on the surface of T cells and APCs. Depending on the type of protein, surface protein–protein interactions can either stimulate or inhibit T cell recruitment induction. T cells are activated when the antigen presented by HLA also interacts with the TCR simultaneously [32,40]. However, cancer is a complex illness in which immunosuppressive cells in the tumor microenvironment, such as regulatory T cells (Treg) and myeloid-derived suppressor cells (MDSCs), moderate the immune response and help cancer cells escape the immune system [41]. Ultimately, the goal of a cancer vaccine is to aggressively activate the CD8+ T cell pathway, which is mediated by CD4+ T cells, overcoming self-tolerance and immune suppression and resulting in cancer cell eradication.

## 4. Cancer Vaccine Candidate Criteria

The specifications of a vaccine depend on the type of vaccine itself. However, all types of vaccines have the same principles: (1) they have sufficient active ingredients to immunize the recipient, (2) they are safe according to regulatory standards, and (3) they have a low level of contamination according to regulatory standards. This principle applies to conventional vaccines and even modern recombinant vaccines [42]. Although it cannot predict the level of contamination, immunoinformatics methods can help in the design process of epitope-based peptide vaccines while still adhering to the other two principles.

Recent advancements in immuno-oncology have demonstrated that manipulating the immune response to oppose the immune evasion strategies used by cancer cells is a potent approach to cancer treatment. A lot of efforts are focused on stimulating T cell responses, because T cells are thought to be capable of clearing malignancies in the absence of immunosuppressive processes [32]. Most cancer cells can be distinguished from healthy cells by either overexpression or mutation of endogenous proteins. Thus, a vaccination target could be any gene product that is made differently or modified in cancer cells compared to in healthy cells.

Both vaccine- and ACT-based immunotherapy strategies require the identification of certain tumor antigens and the T cells that identify them [43]. Regardless of the technique employed, functional assays must be done to confirm the immunogenicity of each newly discovered antigen. This is done by showing that a particular epitope is the only trigger for T cell activation, as opposed to a control (such as a wild-type peptide for mutant antigens) that is attached to the same HLA molecule. It may not always be right to conclude that a peptide is immunogenic simply because it binds to or is projected to bind to an HLA molecule expressed by cancer.

Protein structure analysis is required to determine the structure and physicochemical properties of the target protein. Referring to the lock-and-key interaction model, epitope-based peptide vaccines interact complementarily with immune system components such as antibodies and HLA based on their binding site structure [44]. Therefore, protein structure analysis is important to study how pathogenic proteins interact with the immune system. Protein structure analysis can also show protein stability as a potential vaccine candidate.

Stimulation of the T cell response by the vaccine can be in the form of recruitment of T cells to eradicate pathogens and infected host cells. The antigen-specific T cell response is mediated by the TCR with HLA class I and II. The peptides presented by HLA class I are generally short, although they can still accommodate larger peptides. HLA class I peptides generally follow the X-(L/I)-X(6–7)-(V/L) pattern, where L/I and V/L represent residues whose side chains anchor the peptide to the pHLA, while the other side is attached to the pHLA on the TCR [45]. As for the peptides presented by HLA class II, they are generally more varied in terms of length and sequence but still have anchoring sites. Moreover, for a peptide to be recognized, the peptide presented must have a free N-terminal. These criteria must be met for the peptide to bind to HLA. However, this does not guarantee that the peptides presented will be immunogenic. Thus, at a minimum, a good candidate T cell epitope is an epitope that is antigenic and can bind to several HLA alleles [32]. 

For peptide-based cancer vaccines to work, they need CD8+ epitopes to use the antigen cross-presentation pathway, which activates CTL anti-tumor immunity, and CD4+ epitopes to activate T-helper cells, which keeps the CTL effector function going [46]. As a result, the sequence length of peptide vaccines is critical for eliciting a significant immunogenic response. If the peptide is too short, it could bind to the HLA of non-professional APCs, which do not have the secondary signaling machinery needed for full T cell activation. This could lead to a weak T cell response or immunological tolerance [46]. Shorter peptides are also more likely to be HLA-type restricted because there is not enough variation in the general population for HLA to be very different [46,47]. Lastly, unless they are changed, short peptides are more likely to be broken down by enzymes and leave the body [48,49]. A longer peptide length, on the other hand, provides for larger HLA-type population coverage [46,47], the inclusion of multi-epitope peptides to boost the CD4+ and CD8+ responses, and the presence of binding or recognition motifs to boost immunogenicity. 

The next stage is the characterization of the selected epitopes. The characteristics examined included allergenicity, toxicity, hydrochemical properties, and physiochemical properties. The best epitopes are those that are hypoallergenic, non-toxic, and stable under physiological conditions. 

## 5. Immunoinformatics Approach

**Figure 1:**
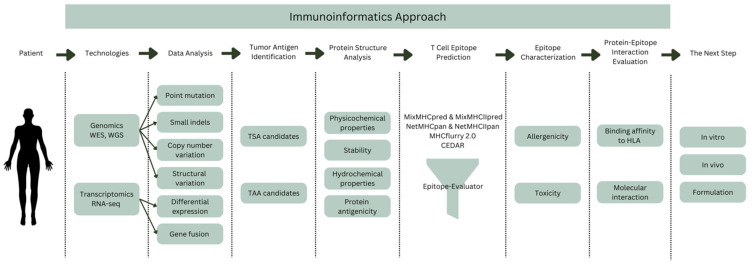
Overview of immunoinformatics approach in cancer vaccine development. Tumor antigens can be identified using high-throughput technologies such as WES, WGS, and RNA-seq. Immunoinformatics can help in protein structure analysis, T cell epitope prediction, epitope characterization, and protein–epitope interaction evaluation. WES—whole exome sequencing; WGS—whole genome sequencing; TSA—tumor-specific antigen, TAA—tumor-associated antigen, HLA—human leukocyte antigen.

Immunoinformatics, often known as computational immunology, is the field that bridges the gap between computer science and experimental immunology. It denotes the use of computational approaches and resources to the comprehension of immunological data. It not only aids in dealing with massive amounts of data, but it also plays an important part in developing novel theories about immune responses. This section will discuss the immunoinformatics approach to cancer vaccine design from tumor antigen identification to protein-epitope interaction evaluation (Figure 1).

### 5.1. Tumor Antigen Identification

Several methods have been developed to predict whether peptides produced from a certain protein, whether wild-type or mutant, are accessible to interact with TCRs on T cells. One such screening method is by analyzing whole-exome sequencing (WES) data from matched tumor and normal DNA to find peptides with tumor-specific non-synonymous mutations. Then, a portion of peptides is created, pumped onto the APCs, and examined for identification by the patient’s own autologous CD8+ T cells. This component of the peptides is projected to bind to patients’ own HLA class I molecules strongly [50].

A variation of this strategy substitutes synthetic multimeric peptide–HLA complexes (such as HLA tetramers) for peptide-pulsed APCs. These complexes are made by combining a variety of fluorescently or genetically tagged HLA molecules and loading them with potential peptides [51]. The enumeration of T lymphocytes that detect potential antigens is made possible by the ability of these complexes to attach to complementary TCRs. This method may work well for finding epitopes that are expected to bind to common class I HLA molecules, but it is not very useful for finding those that bind to class II or less common class I HLA molecules [52]. Additionally, because it involves evaluating peptide libraries linked to specific HLA molecules, this approach frequently falls short of evaluating all potential antigens expressed by the tumor.

In validation trials, however, only a small number of the predicted peptides are found to be immunogenic [53]. Inadequate algorithmic performance with less prevalent class I and most class II HLA molecules, inability to recognize post-translationally changed or spliced peptides, and susceptibility to overlooking some de facto immunogenic peptides are a few of these shortcomings. Various bioinformatical systems additionally employ algorithms that anticipate additional protein or peptide properties implicated in immunogenicity to get around some of these drawbacks. For instance, recent research has combined predictions of peptide–HLA binding affinity, wild-type-over-mutant affinity ratios, and the stability of specific peptide–HLA complexes with information on the expression of cognate genes to create a model with an improved ability to predict immunogenic mutated peptides [54].

To get around the problems with prediction algorithms, another WES-based technique has been developed that allows screening of all possible antigens without limiting the analysis to certain HLA molecules [55]. Metastatic tumors are surgically removed and utilized to establish TIL cultures as well as to perform WES to discover tumor-specific non-synonymous mutations, such as single-nucleotide variations (SNVs) and short (50 bp) insertions and deletions (INDELs). This method will create a peptide pool with tumor-specific mutations. Following that, autologous APCs are pulsed with peptide pools, allowing candidate antigen processing and presentation on all conceivable autologous HLA molecules, and then co-cultured with a panel of TILs. Peptide pools that activate T cells are further deconvoluted to identify tumor antigens. This method does not, however, detect antigens resulting from unmutated genes, gene fusions, abnormal RNA processing, or translation. However, these issues might be solved by using RNA sequencing or whole-genome sequencing (WGS) in a similar tumor-versus-normal approach.

### 5.2. Protein Structure Analysis

Protein structure analysis is carried out to determine the physicochemical properties of the target protein, including GRAVY (grand average of hydropathicity), half-life, molecular weight, stability, and so on, based on the protein’s amino acid sequence. Apart from the primary structure, the secondary and tertiary structures of the protein, the transmembrane topology (if the protein studied is a membrane protein), and the overall antigenicity of the protein need to be analyzed as well. Structural analysis of proteins is important to understand further how the conformation of a protein determines its biological function, in this case, its interaction with the immune system.

Bioinformatics tools capable of predicting this include ProtParam from the ExPASy server. ProtParam (https://web.expasy.org/protparam/, accessed on 12 September 2022) is commonly used to predict the molar exclusion coefficient (also known as molar absorption coefficient), in vivo half-life, instability index, aliphatic index, and GRAVY only with input in the form of amino acid sequences or desired protein ID. A prediction of the molar absorption coefficient can indicate the intensity of light at a certain wavelength that can be absorbed by the protein. This is important in the protein purification process [9].

The half-life of proteins can be estimated by looking at the N groups of the amino acids that make up the protein. A recombinant protein study proved this by testing beta-galactosidase proteins with different N-terminals. The results showed that there was a very sharp difference between the half-lives of these engineered proteins, and even greater differences occurred in different host organisms [56].

Another important characteristic is protein stability. The stability of the protein in the test tube can be predicted by calculating the instability index derived from experimental data. A statistical analysis of 12 unstable proteins and 32 stable proteins showed that there were significant differences in the composition of certain dipeptides in unstable proteins relative to stable proteins [57]. From the results of the analysis, the value of the stability weight of 400 dipeptides was successfully determined and the instability index could be formulated.

Calculating the total hydropathicity value of each amino acid in the sequence divided by the length of the sequence will give us the grand average of hydropathicity (GRAVY) value. There are multiple hydropathicity indexes of each amino acid residue, one of which was proposed by Jack Kyte and Russell F. Doolittle in their experiments. The greater the hydropathicity index, the more hydrophobic the amino acid residue is and vice versa [58].

Furthermore, another bioinformatics tool commonly used in the overall protein antigenicity prediction stage is VaxiJen 2.0 (http://www.ddg-pharmfac.net/vaxijen/VaxiJen/VaxiJen.html, accessed on 12 September 2022). VaxiJen 2.0 is a predictive model of protein antigenicity based on auto cross covariance on the physicochemical properties of proteins (hydrophobicity, spatial arrangement, and polarity). Despite being the older version of VaxiJen, VaxiJen 2.0 can evaluate tumor peptides antigenicity while VaxiJen 3.0 can only target bacteria. It is also independent of sequence alignment, and its accuracy is in the range of 70–89%. Antigen prediction that depends on sequence alignment will be very detrimental for new proteins that may have low similarity to other known proteins [59].

### 5.3. T Cell Epitope Prediction

Vaccines for cancer tend to stimulate T cell recruitment [12,14,15]. Adaptive T cell responses are mediated by TCR and HLA I and II. The choice of T cell epitope is strongly influenced by the sequence, length, and structure of the epitope because the minimum requirement to predict whether a peptide sequence is a candidate epitope for T cells is its ability to bind to HLA A peptide vaccine can also be designed specifically for a particular population by selecting a set of HLA alleles that have high coverage in that population when predicting the T cell epitope.

The IEDB provides T cell epitope prediction services based on binding to HLA with various prediction servers. One that is recommended by the IEDB is the NetMHCpan and NetMHCIIpan servers. NetMHCpan and NetMHCIIpan adopt the NNAlign_MA prediction model [60]. The NNAlign_MA model was trained with binding affinity data and mass spectrometry-eluted HLA ligand data. Mass spectrometry technology is now able to identify the immunopeptidome, which is a group of peptides presented by HLA. Immunopeptidome usually contains multiple sequence motifs that match the HLA molecule that represents it, so this knowledge will be very helpful in the prediction of T cell epitopes. NNAlign_MA has been benchmarked on a large and diverse dataset, including data from HLA class I and II. The NNAlign_MA performance assay successfully surpassed other T cell epitope prediction methods. Another advantage of NNAlign_MA is its ability to widen the range of alleles, so predictions can be made more accurately. It also improves the quality of T cell epitope identification [60]. The service delivers the probability of a peptide being a natural ligand of the given MHC(s) as the default. If chosen, the expected binding affinity is also provided [61].

Another prediction service to study HLA class I-presented peptides is MHCflurry 2.0 [62]. MHCflurry 2.0 employs different predictors for MHC allele-dependent and allele-independent effects (binding affinity (BA) prediction and antigen processing (AP) prediction). MHCflurry 2.0 BA, a novel pan-allele MHC class I BA predictor, was initially trained using accessible MHC class I ligand data, including affinity measurements and MS datasets. One of numerous design choices designed to reduce the BA predictor’s ability to learn AP signals is the inclusion of in vitro affinity measurements in the training data, which are essentially independent of AP. The BA predictor is then used to construct a training set for an AP model by mixing MS-identified peptides (hits) with unobserved peptides (decoys), where the BA predictor predicts that both hits and decoys will bind the corresponding HLA class I alleles. The AP predictor thereby predicts the remaining allele-independent sequence features that the BA predictor did not learn. The processing predictor preferred sequences congruent with recognized patterns for essential AP stages and demonstrated quantitative agreement with an independent dataset of proteasome-cleaved peptides, demonstrating its biological significance [63]. MHCflurry 2.0 took the BA and AP variables and put them together in a logistic regression model to get the presentation score (PS).

Aside from those webtools, the Ludwig Institute for Cancer Research developed command-line programs to predict T cell epitopes called MixMHCpred and MixMHC2pred. MixMHCpred can rapidly identify many HLA-I binding motifs and map them to their corresponding alleles without any prior knowledge of HLA-I binding specificity. MixMHCpred was trained using the co-occurrence of HLA-I alleles across ten newly generated as well as forty public HLA peptidomics datasets containing more than 115,000 unique peptides [64]. For the HLA-II counterparts, MixMHC2pred was trained using unbiased mass spectrometry-based HLA-II peptidomics with a novel motif deconvolution algorithm to profile and analyze a total of 99,265 unique HLA-II ligands [65]. These two predictors’ training data is very physiologically relevant, making them more suited for predicting HLA presentation.

To some, command-line programs may seem intimidating, and some may say the results are not straightforward. An alternative that provides a user-friendly interface is the Epitope-Evaluator [66]. The Epitope-Evaluator makes it easier to analyze, visualize, and filter the predicted epitopes interactively. It includes six tools for doing a thorough epitope analysis, including interactive graphs and downloadable findings. These tools can be used for a variety of biological applications, such as identifying proteins and regions to design peptide-based vaccines, identifying promiscuous and conserved epitopes for the development of multi-epitope vaccines, and studying the impact of mutations on the formation of neo-epitopes. Although Epitope-Evaluator examines epitope binding strength, position, and other sequence-based criteria, other elements connected with each protein’s biological function must be addressed for rational vaccine formulation. Because these factors are case-specific, Epitope-Evaluator does not take them into account.

Benchmarking reports show comparable results for the predictors mentioned. Zhao et al. [67] tested those predictors on an extensive set of MHC-binding predictors by using newly available, untested data for both synthetic and naturally processed epitopes. Algorithm-wise, predictors based on artificial neural networks (ANN) outperformed regression-based machine learning and structural modeling even though they delivered low correlations between the predicted and experimental affinities for strong MHC binders. When employed on naturally processed MHC ligands, algorithms trained on elution data (NetMHCpan4 and MixMHCpred) outperform predictors that utilize binding affinity only.

Another benchmarking report by Mei et al. [68] indicates that MixMHCpred 2.0.1 outperforms the other machine learning- and consensus-based tools for predicting peptides’ binding to most of the HLA-I allomorphs tested, while NetMHCpan 4.0 and NetMHCcons 1.1 beat the other machine learning- and consensus-based methods. It is essential to mention that a peptide with a higher binding score for a given HLA allotype does not always mean that it would be immunogenic. Nonetheless, peptide-binding predictors can help drastically minimize the vast number of epitope possibilities that must be experimentally validated.

All the tools mentioned above are meant for generalized T cell epitope predictions, including pathogens, allergies, transplantation, and autoimmunity. Recently, a new specialized cancer epitope database has been developed to fill the gaps in cancer epitope prediction. The Cancer Epitope Database and Analysis Resource (CEDAR) [69] is envisioned as a complete bioinformatics resource that will give access to curated cancer epitope data, including mutant and non-mutated cancer epitopes, as well as bioinformatics tools for epitope and receptor research and prediction. The planned effort would expand on the IEDB. CEDAR, like the IEDB, will incorporate all cancer-specific epitope data from multiple T and B cell investigations, MHC binding tests, and mass spectrometry-based MHC ligandomics. CEDAR will also provide in vivo experiment outcomes such as tumor rejection and/or tumor control information. With CEDAR’s fine-grained data curation and flexible query structure, the users will be able to run many queries to find epitopes that are supported by different experimental data. CEDAR returns query results in three different formats: (i) tables on the results homepage that provide important values such as host and assay type, as well as summaries of more complicated data such as immunization fields; (ii) assay details pages that present the majority of areas provided and details pages for epitopes with information on the epitope and links to all tests; (iii) spreadsheet exports of results that have a large number of data field columns, whether filled or not. CEDAR creates a results page tab to present a summary table of the receptor sequences relevant to the search criteria as the receptor sequence data are updated. Similarly, additional receptor information pages and an export table with this data will be created.

### 5.4. Epitope Characterization

Epitope prediction results only look at the antigenicity of the candidate epitope. However, all medical interventions are expected to be safe to apply to the target subject. To eliminate the negative possibilities that can occur during vaccine administration, it is necessary to characterize the candidate epitope. The characteristics that are generally examined include allergenicity, toxicity, hydro-chemical properties, and physicochemical properties. The prediction of hydrochemical and physicochemical properties has been described previously. This section will focus more on predicting allergenicity and toxicity.

To predict allergenicity, one of the tools that can be used is AllergenFP 1.0 (http://ddg-pharmfac.net/AllergenFP/, accessed on 13 September 2022). AllergenFP 1.0 already has a dataset of allergens and non-allergens. AllergenFP 1.0 uses auto cross covariance (ACC) to generalize the length of all peptides. The analyzed epitopes will be compared by calculating the Tanimoto coefficient. If the epitope has a Tanimoto coefficient that is closer to the allergen data, then the epitope is predicted to be an allergen and vice versa [70].

Meanwhile, to predict the toxicity of the epitopes, one can use ToxinPred (https://webs.iiitd.edu.in/raghava/toxinpred/index.html, accessed on 13 September 2022). ToxinPred uses a dipeptide-based support vector machine (SVM) machine learning technique to obtain a toxicity prediction model. The training data used by the SVM model contained 1805 toxic peptides having 35 residues and a total of 3593 non-toxic peptides from SwissProt and TrEMBL. Residues such as Cys, His, Asn, and Pro were observed to be abundant in toxic peptides. The performance of the dipeptide base model has an accuracy of 94.5%. Another model available in ToxinPred apart from the dipeptide-based SVM model is the hybrid model, which combines the SVM model with the search for previously known toxic protein motifs. If a toxic protein motif is found in the query, then the results of the SVM model are increased by 5. This hybrid model turns out to have a better accuracy, which is 98.41% [71].

### 5.5. Protein–Epitope Interaction Evaluation

Epitopes that have a high binding affinity with alleles that have been experimentally validated will be strong epitope candidates. Therefore, many in silico vaccine design studies have also tested the binding of epitopes to human alleles using the molecular docking method. Molecular docking is an in-silico method that is applied to model protein–ligand interactions at the atomic level. This modeling helps characterize the behavior of ligands at binding sites on target proteins [72]. Molecular docking can show the binding affinity and important residues involved in the interaction, as well as the type of interaction.

There are few approaches to dock a peptide to a receptor. One can do molecular docking through comparative approaches by constructing a model of the complex using known structures (templates) as scaffolds. This strategy can be very useful if the template is similar to the complex under investigation. The GalaxyPepDock web server uses an automated template-based method. It looks for templates based on similarities between the input protein structure, protein–peptide interaction, and complex structures contained in the PDB [73]. Then, it constructs complex models using energy-based optimization and refinement, allowing for structural flexibility. With the FlexPepBind method, for example, it allows for the modeling of various peptide sequences into receptor binding sites, with limitations that reinforce certain key properties such as preserved hydrogen bonds [74].

Some molecular docking software uses a local docking approach. Local docking approaches look for peptide binding poses in the vicinity of a user-defined binding site; hence, docking accuracy is dependent on the binding site input information: the more accurate, the better. The offered approaches define the binding location in various ways. Rosetta FlexPepDock [75] and DynaDock [76] ask the user to provide an initial model of the complex. As proven, the approaches should be able to enhance the original model if its accuracy is within a 5 backbone root mean square deviation (RMSD) of the experimental structure. Furthermore, the input model may require method-specific preparation, such as the removal of internal conflicts [74]. Some of the approaches, however, need less tightly stated beginning models. Because the approach allows for considerable peptide flexibility and broad sampling of rigid body orientations inside the binding site, the input peptide conformation in Rosetta FlexPepDock ab initio [77] may be distant from the native. HADDOCK [78], on the other hand, may automatically position the peptide at the binding site determined by a user-supplied list of interface residues. AutoDock Vina is a standalone software alternative that employs local docking, though it is limited to short peptides only [79].

Alternatively, global docking approaches look for the peptide binding location and posture simultaneously. The most basic method for global protein–peptide docking is to consider the protein and peptide input conformations stiff and execute thorough rigid-body docking. More advanced algorithms anticipate peptide conformation using a user-supplied sequence. Their pipelines typically consist of three stages: (i) creation of input peptide conformations; (ii) rigid-body docking; and (iii) scoring and/or refining of the models. Various methodologies can be used to predict the peptide conformation (e.g., using structure fragments from monomeric protein structures [80], threading the sequence onto a predefined set of template conformations [81], or simulating peptide folding in the solution [82]). Peptide conformation generation can also be integrated with global docking in a single explicit simulation. This is achievable in the CABS-dock technique [83], which starts with random shapes for the peptides and only changes when they interact with a flexible receptor. Alternatively, global docking can be paired with binding site predictions. This method is employed in AnchorDock [82], which automatically detects possible binding sites and docks a flexible peptide in their vicinity.

## 6. The Next Step

Although all the immunoinformatics methods described above have a strong statistical basis and use experimental data as training data for predictive models, these models still have biases that should not be ignored. This bias can lead to errors in predictions. Therefore, predictive models always have values of accuracy, sensitivity, specificity, and so on to benchmark the reliability of the prediction results. In addition, many physiological mechanisms are not well modeled in immunoinformatics methods. Many factors can make the prediction model deviate from its true value. Furthermore, there is no predictive model that can predict the response of T cells after activation. Once activated, CD4+ T cells, for example, mature into Th1 cells or Th2 cells. The typical Th1 cytokine is IFN-ℽ, whereas Th2 has the typical cytokines IL-4, IL-5, and IL-10 [84]. However, the change in T cell response is determined by costimulatory signals, TLR, and PAMPS activation, not epitope. Several studies have reported a change in the type of immune response when an amino acid in the epitope is changed [85,86]. Therefore, more research needs to be done in vitro and in vivo to confirm the predicted results.

It has been explained previously that the vaccine design must meet at least three things: (1) have sufficient active ingredients to immunize the recipient; (2) have a safety level per regulatory standards; and (3) have a low level of contamination according to regulatory standards. In silico studies have been able to predict the first and second points. Thus, in addition to confirming the predicted results obtained, further studies should be able to confirm these three things through in vitro and in vivo tests.

In vitro and in vivo testing of such vaccine designs usually begins with synthesizing peptide vaccine candidates. Generally, peptides can be synthesized in two ways: soluble phase synthesis and solid phase synthesis. One well-known strategy is the Merrifield solid-phase protein synthesis method [87]. After synthesis, peptides need to be characterized and purified. The characterization method that is often used is mass spectroscopy. Other methods, such as FT-IR spectroscopy, can also be performed to determine the functional characteristics of peptides [88]. The 2D and 3D structures of peptides can be analyzed using CD spectroscopy [89] and many other characterization methods. As for the purification of peptides, the HPLC method is more widely used. Ion exchange chromatography and affinity chromatography can also be performed for the purification of certain peptides [90,91].

Epitope-based peptide vaccine designs were forced to compromise efficacy because epitope is a short peptide sequence. Thus, it is less antigenic than other vaccines and even easily disappears in the body because of its small size. Further studies should also consider adding suitable adjuvants to epitope-based peptide vaccines to enhance antigen immunogenicity. There are two types of adjuvants that can be used for epitope-based peptide vaccines, namely, immunostimulants and carrier molecules that can regulate epitope delivery and release. Currently, there is no consensus regarding the most optimal adjuvant to be used for a given peptide vaccine, and this could be a promising research area to further optimize and improve vaccine formulations [92]. All phase I and II peptide-based cancer vaccine studies that are actively active or recruiting participants include Montanide ISA-51, GM-CSF, poly-ICLC, and many more [93].

In the case of breast cancer, one of the most well-known TAAs is HER2. There are a lot of vaccines that target HER2-related antigens. One of them is E75, a breast cancer vaccine that uses the immunologic adjuvant GM-CSF to target the HLA-A2/A3-restricted, HLA class-I, extracellular HER2-derived peptide E75.It is one of the most studied epitope-based vaccines that was identified in 1995 [94]. The E75 vaccine was provided to disease-free patients with any degree of HER2 expression (immunohistochemistry (IHC) 1–3+) in a phase 1 adjuvant study. An immunological response with high tolerance was seen [95]. It was established that a monthly intradermal dosage of 1000 mg E75 and 250 mg GM-CSF for 6 months was best [96]. In the subsequent phase 2 trial, 195 patients were randomly allocated to either the vaccine or control arm. At the end of a 5-year follow-up, the disease-free survival (DFS) rate for vaccinated individuals was 89.7% against 80.2% for control patients (*p* = 0.08) [96,97]. Interestingly, vaccinated individuals with relatively low HER2 expression (IHC 1–2+) displayed a more robust immunological response than those with greater levels of HER2 expression (IHC 3+), indicating that immunologic tolerance to HER2 may exist in certain patients with tumors expressing high levels of HER2 [23].

In a recently completed phase 2 adjuvant study, the efficacy of the E75 vaccination in patients with low HER2 expression (IHC 1–2+) when combined with anti-HER2-targeted treatment was evaluated [98]. Following a year of conventional trastuzumab-based anti-HER2 therapy, 275 patients were randomly assigned to receive E75 or a placebo. Estimated DFS did not change substantially between the vaccine and control arms at a median follow-up of 25.7 months (*p* = 0.18). In a planned exploratory study, however, individuals with TNBC had significantly better DFS (*p* = 0.01). This study suggests that HER2-derived peptide vaccines may be efficacious when administered in conjunction with or in addition to trastuzumab-based anti-HER2-targeted treatment. In the case of HER2 overexpression (IHC 3+) patients, the efficacy of E75 remains unclear because the majority of HER2 overexpression patients who participated in prior trials did not get trastuzumab as conventional anti-HER2 treatment.

GP2 is another HLA class-I, HLA-A2/A3-restricted immunogenic peptide generated from the transmembrane region of HER2. GP2 has a lesser affinity for HLA-A2 than E75, but it is just as effective at inducing a CD8+ T cell response [24]. In a phase 1 adjuvant experiment, the GP2 vaccination displayed a satisfactory safety profile and generated GP2-specific T cell responses as well as GP2-specific delayed-type hypersensitivity (DTH) [99]. After a 34-month median follow-up in the following phase 2 adjuvant study, which involved 180 patients with tumors expressing HER2 (IHC 1–3+), there was no significant advantage in DFS in the vaccine group compared to the control group (88% vs. 81%, *p* = 0.43) [100]. A subgroup analysis revealed that HER2-positive (IHC 3+) patients had no recurrences, with a tendency toward better DFS in the vaccine group than the control group (100% vs. 87.2%, *p* = 0.052) [25]. The final analysis of this experiment revealed encouraging findings, demonstrating that the GP2 vaccination lowered the recurrence rate to 0% in HER 3+ patients who had a conventional course of trastuzumab following surgery. If the patient completed the main vaccination series, the projected 5-year DFS rate in the 46 HER2 3+ vaccinated individuals was 100% vs 89.4% in the 50 placebo patients (*p* = 0.034) [101].

Aside from E75 and GP2, another HER2-related peptide vaccination utilized in the adjuvant context of breast cancer is AE37. It is an Ii-Key hybrid of AE36 generated from the HER2 intracellular domain. The alteration was carried out to boost the epitope’s binding efficacy [102]. Unlike E75 and GP2, AE37 is an HLA class-II epitope that primarily activates CD4+ T cells. A phase 1 experiment revealed little toxicity and a positive immunological response [103]. Treg cell levels were evaluated and found to be lower after immunization because AE37 promotes a CD4+ helper T cell response [103]. In a phase 2 study, 153 patients got AE37 with GM-CSF and 145 patients got GM-CSF alone. Both groups were made up of clinically healthy people with any amount of HER2 (IHC 1–3+) [104]. After a median of 30 months, the DFS rate in the vaccine group was 87.6% and 86.2% in the control group (*p* = 0.70). DFS was 86.8% in vaccinated individuals and 82.0% in control patients in a planned subset analysis of patients with IHC 1–2+ HER2-expressing tumors (*p* = 0.21). TNBC patients (IHC 1–2+ and hormone receptor-negative) had a DFS rate of 84.0% in the vaccine group and 64.0% in the control group (*p* = 0.12), indicating that AE37 immunization may provide therapeutic advantages in patients with low HER2-expressing malignancies, especially TNBC.

As for TSA-based vaccines, it has been mentioned before that the fundamental limitation of cancer vaccines based on neoantigens is that they are highly personalized, and their identification requires a combination of high-throughput genomes, proteomics, and immunomics screening tools that are currently not widely available. Moreover, the efficacy of such a highly personalized method may be limited by the fast mutational rate of tumors, which results in the continuous generation of new target mutated neoantigens and, as a result, cancer immune evasion. Only a couple of breast cancer vaccines in clinical trials are based on TSAs; one of them is atezolizumab neoantigen vaccine (NCT132289962) and the other is a combined therapy of durvalumab, Nab-paclitaxel, and neoantigen (NCT03606967) [22,105].

Moving forward, we can expect that cancer treatment will become more personalized as the technology advances. There is also great potential that breast cancer vaccines may improve the outcome when combined with other therapies. Researchers are studying the efficacy and safety of vaccine-combined therapies for cancer [106,107,108,109,110]. An anti-HER-2 monoclonal antibody used to treat breast cancer, trastuzumab, was discovered to make HER-2-positive tumor cells more vulnerable to antibody-dependent and T cell-mediated cytotoxicity [111,112]. Gall et al. discovered that trastuzumab increased DC absorption and cross-presentation of HER-2-derived peptides (E75), resulting in anticancer immune priming and increased generation of antigen-specific CTLs [113]. Furthermore, in a phase IIb clinical trial, the combination of trastuzumab with GM-CSF and E75 peptide (nelipepimut-S) was proven to be safe with no increased harm compared to trastuzumab alone, even after prolonged exposure. There was no significant difference in disease-free survival in HER-2 low-expressing breast cancer, but there was a substantial clinical advantage in triple-negative breast cancer (TNBC) patients [98,114]. These findings imply that a combination of nelipepimut-S and trastuzumab might be employed as adjuvant treatment for early TNBC and justify further investigation in phase III randomized trials.

## 7. Conclusions

The immunoinformatics approach to epitope-based peptide vaccine design has great potential for helping accelerate vaccine development. The immunoinformatics method can provide more comprehensive data and information about vaccine candidates compared to conventional methods. These data are very helpful in designing more specific vaccines. TAAs are useful for producing a single vaccination that can be made in huge quantities and disseminated to many patients as a one-for-all strategy. One of the most difficult issues is ensuring that TAAs elicit the optimal immune response. The immune system is meticulously calibrated to ensure that it does not harm the body. Meanwhile, the main constraint of cancer vaccines based on altered neoantigens is that they are strictly personalised, and their discovery necessitates a combination of high-throughput genomics, proteomics, and immunomics screening technologies that are presently not applicable on a broad scale. One of the most important characteristics of an epitope-based vaccine is that it needs to properly trigger an immune response. T cells are activated when the antigen presented by HLA also interacts with the TCR simultaneously. There is a lot of immunoinformatics software or techniques that can help with tumor antigen identification, protein structure analysis, T cell epitope prediction, epitope characterization, and protein–peptide interaction evaluation. We discussed many immunoinformatics tools that can help each step of developing cancer vaccines. The low immunogenicity of the epitope is the biggest obstacle to the development of epitope-based peptide vaccines. However, the addition of adjuvants can be a solution to overcome this. Combinatorial therapies of cancer vaccines with anti-cancer drugs give hopeful results as well. As the technology advances, we can hope that cancer vaccines will become more personalized and targeted in the future.

## References

[1] Observatory G. (2020). Breast Fact Sheet.

[2] Gautama W. (2022). Breast Cancer in Indonesia in 2022: 30 Years of Marching in Place. Indones. J. Cancer.

[3] Arnold M., Morgan E., Rumgay H., Mafra A., Singh D., Laversanne M., Vignat J., Gralow J.R., Cardoso F., Siesling S. (2022). Current and Future Burden of Breast Cancer: Global Statistics for 2020 and 2040. Breast.

[4] Behravan J., Razazan A., Behravan G. (2019). Towards Breast Cancer Vaccines, Progress and Challenges. Curr. Drug Discov. Technol..

[5] McKittrick G., Shepherd P., Gilleece T. (2021). Management of Breast Cancer: An Overview for Therapeutic Radiographers. J. Radiother. Pract..

[6] Fadilah F., Erlina L., Paramita R.I., Istiadi K.A. (2021). Immunoinformatics Studies and Design of Breast Cancer Multiepitope Peptide Vaccines: Diversity Analysis Approach. J. Appl. Pharm. Sci..

[7] Abdelmoneim A.H., Mustafa M.I., Abdelmageed M.I., Murshed N.S., Dawoud E.D., Ahmed E.M., Kamal Eldein R.M., Elfadol N.M., Sati A.O.M., Makhawi A.M. (2020). Immunoinformatics Design of Multiepitopes Peptide-Based Universal Cancer Vaccine Using Matrix Metalloproteinase-9 Protein as a Target. Immunol. Med..

[8] Fleri W., Paul S., Dhanda S.K., Mahajan S., Xu X., Peters B., Sette A. (2017). The Immune Epitope Database and Analysis Resource in Epitope Discovery and Synthetic Vaccine Design. Front. Immunol..

[9] Kanampalliwar A.M., Soni R., Girdhar A., Tiwari A. (2013). Reverse Vaccinology: Basics and Applications. J. Vaccines Vaccin.

[10] Liu J., Fu M., Wang M., Wan D., Wei Y., Wei X. (2022). Cancer Vaccines as Promising Immuno-Therapeutics: Platforms and Current Progress. J. Hematol. Oncol..

[11] Graham D.B., Luo C., O’Connell D.J., Lefkovith A., Brown E.M., Yassour M., Varma M., Abelin J.G., Conway K.L., Jasso G.J. (2018). Antigen Discovery and Specification of Immunodominance Hierarchies for MHCII-Restricted Epitopes. Nat. Med..

[12] Tay R.E., Richardson E.K., Toh H.C. (2020). Revisiting the Role of CD4+ T Cells in Cancer Immunotherapy—New Insights into Old Paradigms. Cancer Gene Ther..

[13] van der Burg Franken H., Cornelis J.M., Melief R., Offringa S., Martijn S., Bijker S.J.F., van den Eeden K.L. (2007). CD8+ CTL Priming by Exact Peptide Epitopes in Incomplete Freund’s Adjuvant Induces a Vanishing CTL Response, Whereas Long Peptides Induce Sustained CTL Reactivity. J. Immunol..

[14] Bijker M.S., van den Eeden S.J.F., Franken K.L., Melief C.J.M., van der Burg S.H., Offringa R. (2008). Superior Induction of Anti-Tumor CTL Immunity by Extended Peptide Vaccines Involves Prolonged, DC-Focused Antigen Presentation. Eur. J. Immunol..

[15] Yang J.C., Rosenberg S.A. (2016). Adoptive T-Cell Therapy for Cancer. Adv. Immunol..

[16] Jhunjhunwala S., Hammer C., Delamarre L. (2021). Antigen Presentation in Cancer: Insights into Tumour Immunogenicity and Immune Evasion. Nat. Rev. Cancer.

[17] Radford K.J., Higgins D.E., Pasquini S., Cheadle E.J., Carta L., Jackson A.M., Lemoine N.R., Vassaux G. (2002). A Recombinant *E. coli* Vaccine to Promote MHC Class I-Dependent Antigen Presentation: Application to Cancer Immunotherapy. Gene.

[18] Wong-Arce A., González-Ortega O., Rosales-Mendoza S. (2017). Plant-Made Vaccines in the Fight Against Cancer. Trends Biotechnol..

[19] Heery C.R., Singh B.H., Rauckhorst M., Marté J.L., Donahue R.N., Grenga I., Rodell T.C., Dahut W., Arlen P.M., Madan R.A. (2015). Phase I Trial of a Yeast-Based Therapeutic Cancer Vaccine (GI-6301) Targeting the Transcription Factor Brachyury. Cancer Immunol. Res..

[20] Betting D.J., Mu X.Y., Kafi K., McDonnel D., Rosas F., Gold D.P., Timmerman J.M. (2009). Enhanced Immune Stimulation by a Therapeutic Lymphoma Tumor Antigen Vaccine Produced in Insect Cells Involves Mannose Receptor Targeting to Antigen Presenting Cells. Vaccine.

[21] Song C., Zheng X.J., Liu C.C., Zhou Y., Ye X.S. (2017). A Cancer Vaccine Based on Fluorine-Modified Sialyl-Tn Induces Robust Immune Responses in a Murine Model. Oncotarget.

[22] Buonaguro L., Tagliamonte M. (2020). Selecting Target Antigens for Cancer Vaccine Development. Vaccines.

[23] Benavides L.C., Gates J.D., Carmichael M.G., Patel R., Holmes J.P., Hueman M.T., Mittendorf E.A., Craig D., Stojadinovic A., Ponniah S. (2009). The Impact of HER2/Neu Expression Level on Response to the E75 Vaccine: From U.S. Military Cancer Institute Clinical Trials Group Study I-01 and I-02. Clin. Cancer Res..

[24] Mittendorf E.A., Storrer C.E., Foley R.J., Harris K., Jama Y., Shriver C.D., Ponniah S., Peoples G.E. (2006). Evaluation of the HER2/Neu-Derived Peptide GP2 for Use in a Peptide-Based Breast Cancer Vaccine Trial. Cancer.

[25] Brown T.A., Mittendorf E.A., Hale D.F., Myers J.W., Peace K.M., Jackson D.O., Greene J.M., Vreeland T.J., Clifton G.T., Ardavanis A. (2020). Prospective, Randomized, Single-Blinded, Multi-Center Phase II Trial of Two HER2 Peptide Vaccines, GP2 and AE37, in Breast Cancer Patients to Prevent Recurrence. Breast Cancer Res. Treat..

[26] Schumacher T.N., Scheper W., Kvistborg P. (2018). Cancer Neoantigens. Annu. Rev. Immunol..

[27] Yarchoan M., Johnson B.A., Lutz E.R., Laheru D.A., Jaffee E.M. (2017). Targeting Neoantigens to Augment Antitumour Immunity. Nat. Rev. Cancer.

[28] Schumacher T.N., Schreiber R.D. (2015). Neoantigens in Cancer Immunotherapy. Science.

[29] Reddehase M.J., Rothbard J.B., Koszinowski U.H. (1989). A Pentapeptide as Minimal Antigenic Determinant for MHC Class I-Restricted T Lymphocytes. Nature.

[30] Hemmer B., Kondo T., Gran B., Pinilla C., Cortese I., Pascal J., Tzou A., McFarland H.F., Houghten R., Martin R. (2000). Minimal Peptide Length Requirements for CD4+ T Cell Clones—Implications for Molecular Mimicry and T Cell Survival. Int. Immunol..

[31] Fishman J.M., Wiles K., Wood K.J. (2015). The Acquired Immune System Response to Biomaterials, Including Both Naturally Occurring and Synthetic Biomaterials. Host Response to Biomaterials: The Impact of Host Response on Biomaterial Selection.

[32] Malonis R.J., Lai J.R., Vergnolle O. (2020). Peptide-Based Vaccines: Current Progress and Future Challenges. Chem. Rev..

[33] Chowell D., Morris L.G.T., Grigg C.M., Weber J.K., Samstein R.M., Makarov V., Kuo F., Kendall S.M., Requena D., Riaz N. (2018). Patient HLA Class I Genotype Influences Cancer Response to Checkpoint Blockade Immunotherapy. Science.

[34] Bui H.-H., Sidney J., Dinh K., Southwood S., Newman M.J., Sette A. (2006). Predicting Population Coverage of T-Cell Epitope-Based Diagnostics and Vaccines. BMC Bioinform..

[35] Gonzalez-Galarza F.F., McCabe A., Melo dos Santos E.J., Jones A.R., Middleton D. (2021). A Snapshot of Human Leukocyte Antigen (HLA) Diversity Using Data from the Allele Frequency Net Database. Hum. Immunol..

[36] Gonzalez-Galarza F.F., McCabe A., dos Santos E.J.M., Jones J., Takeshita L., Ortega-Rivera N.D., Cid-Pavon G.M.D., Ramsbottom K., Ghattaoraya G., Alfirevic A. (2020). Allele Frequency Net Database (AFND) 2020 Update: Gold-Standard Data Classification, Open Access Genotype Data and New Query Tools. Nucleic Acids Res..

[37] Martini S., Nielsen M., Peters B., Sette A. (2020). The Immune Epitope Database and Analysis Resource Program 2003–2018: Reflections and Outlook. Immunogenetics.

[38] Requena D., Médico A., Chacón R.D., Ramírez M., Marín-Sánchez O. (2020). Identification of Novel Candidate Epitopes on SARS-CoV-2 Proteins for South America: A Review of HLA Frequencies by Country. Front. Immunol..

[39] Janse van Rensburg W.J., de Kock A., Bester C., Kloppers J.F. (2021). HLA Major Allele Group Frequencies in a Diverse Population of the Free State Province, South Africa. Heliyon.

[40] Lin M.J., Svensson-Arvelund J., Lubitz G.S., Marabelle A., Melero I., Brown B.D., Brody J.D. (2022). Cancer Vaccines: The next Immunotherapy Frontier. Nat. Cancer.

[41] Hatziioannou A., Alissafi T., Verginis P. (2017). Myeloid-Derived Suppressor Cells and T Regulatory Cells in Tumors: Unraveling the Dark Side of the Force. J. Leukoc. Biol..

[42] Minor P. (2012). Considerations for Setting the Specifications of Vaccines. Expert Rev. Vaccines.

[43] Wang M., Yin B., Wang H.Y., Wang R.F. (2014). Current Advances in T-Cell-Based Cancer Immunotherapy. Immunotherapy.

[44] Braden B.C., Dall’Acqua W., Eisenstein E., Fields B.A., Goldbaum F.A., Malchiodi E.L., Mariuzza R.A., Schwarz F.P., Ysern X., Poljak R.J. (1995). Protein Motion and Lock and Key Complementarity in Antigen-Antibody Reactions. Pharm. Acta Helv..

[45] Garboczi D.N., Ghosh P., Utz U., Fan Q.R., Biddison W.E., Wiley D.C. (1996). Structure of the Complex between Human T-Cell Receptor, Viral Peptide and HLA-A2. Nature.

[46] Sidney J., del Guercio M.F., Southwood S., Engelhard V.H., Appella E., Rammensee H.G., Falk K., Rötzschke O., Takiguchi M., Kubo R.T. (1995). Several HLA Alleles Share Overlapping Peptide Specificities. J. Immunol..

[47] Chesnut R.W., Grey H.M., Guercio A.S., Appella E., Hoffman S., Kubo R.T., Southwood S., Sidney J., Kondo A., Del M.-F. (2022). Overlapping Peptide Binding Repertoires Several Common HLA-DR Types Share Largely. J. Immunol. Ref..

[48] Diao L., Meibohm B. (2013). Pharmacokinetics and Pharmacokinetic–Pharmacodynamic Correlations of Therapeutic Peptides. Clin. Pharmacokinet..

[49] Di L. (2015). Strategic Approaches to Optimizing Peptide ADME Properties. AAPS J..

[50] Robbins P.F., Lu Y.C., El-Gamil M., Li Y.F., Gross C., Gartner J., Lin J.C., Teer J.K., Cliften P., Tycksen E. (2013). Mining Exomic Sequencing Data to Identify Mutated Antigens Recognized by Adoptively Transferred Tumor-Reactive T Cells. Nat. Med..

[51] Cohen C.J., Gartner J.J., Horovitz-Fried M., Shamalov K., Trebska-McGowan K., Bliskovsky V.V., Parkhurst M.R., Ankri C., Prickett T.D., Crystal J.S. (2015). Isolation of Neoantigen-Specific T Cells from Tumor and Peripheral Lymphocytes. J. Clin. Investig..

[52] Vollers S.S., Stern L.J. (2008). Class II Major Histocompatibility Complex Tetramer Staining: Progress, Problems, and Prospects. Immunology.

[53] Garcia-Garijo A., Fajardo C.A., Gros A. (2019). Determinants for Neoantigen Identification. Front. Immunol..

[54] Koşaloğlu-Yalçın Z., Lanka M., Frentzen A., Logandha Ramamoorthy Premlal A., Sidney J., Vaughan K., Greenbaum J., Robbins P., Gartner J., Sette A. (2018). Predicting T Cell Recognition of MHC Class I Restricted Neoepitopes. Oncoimmunology.

[55] Tran E., Robbins P.F., Rosenberg S.A. (2017). ‘Final Common Pathway’ of Human Cancer Immunotherapy: Targeting Random Somatic Mutations. Nat. Immunol..

[56] Varshavsky A. (1997). The N-End Rule Pathway of Protein Degradation. Genes Cells.

[57] Guruprasad K., Reddy B.V.B., Pandit M.W. (1990). Correlation between Stability of a Protein and Its Dipeptide Composition: A Novel Approach for Predicting in Vivo Stability of a Protein from Its Primary Sequence. Protein Eng. Des. Sel..

[58] Kyte J., Doolittle R.F. (1982). A Simple Method for Displaying the Hydropathic Character of a Protein. J. Mol. Biol..

[59] Doytchinova I.A., Flower D.R. (2007). VaxiJen: A Server for Prediction of Protective Antigens, Tumour Antigens and Subunit Vaccines. BMC Bioinform..

[60] Alvarez B., Reynisson B., Barra C., Buus S., Ternette N., Connelley T., Andreatta M., Nielsen M. (2019). NNAlign_MA; MHC Peptidome Deconvolution for Accurate MHC Binding Motif Characterization and Improved T-Cell Epitope Predictions. Mol. Cell. Proteom..

[61] Reynisson B., Alvarez B., Paul S., Peters B., Nielsen M. (2020). NetMHCpan-4.1 and NetMHCIIpan-4.0: Improved Predictions of MHC Antigen Presentation by Concurrent Motif Deconvolution and Integration of MS MHC Eluted Ligand Data. Nucleic Acids Res..

[62] O’Donnell T.J., Rubinsteyn A., Laserson U. (2020). MHCflurry 2.0: Improved Pan-Allele Prediction of MHC Class I-Presented Peptides by Incorporating Antigen Processing. Cell Syst..

[63] Wolf-Levy H., Javitt A., Eisenberg-Lerner A., Kacen A., Ulman A., Sheban D., Dassa B., Fishbain-Yoskovitz V., Carmona-Rivera C., Kramer M.P. (2018). Revealing the Cellular Degradome by Mass Spectrometry Analysis of Proteasome-Cleaved Peptides. Nat. Biotechnol..

[64] Bassani-Sternberg M., Chong C., Guillaume P., Solleder M., Pak H.S., Gannon P.O., Kandalaft L.E., Coukos G., Gfeller D. (2017). Deciphering HLA-I Motifs across HLA Peptidomes Improves Neo-Antigen Predictions and Identifies Allostery Regulating HLA Specificity. PLoS Comput. Biol..

[65] Racle J., Michaux J., Rockinger G.A., Arnaud M., Bobisse S., Chong C., Guillaume P., Coukos G., Harari A., Jandus C. (2019). HLA-II Motif Deconvolution for Robust Epitope Predictions Deep Motif Deconvolution of HLA-II Peptidomes for Robust Class II Epitope Predictions Running Title: HLA-II Motif Deconvolution for Robust Epitope Predictions. bioRxiv.

[66] Soto L.F., Requena D., Fuxman Bass J.I. (2022). Epitope-Evaluator: An Interactive Web Application to Study Predicted T-Cell Epitopes. PLoS ONE.

[67] Zhao W., Sher X. (2018). Systematically Benchmarking Peptide-MHC Binding Predictors: From Synthetic to Naturally Processed Epitopes. PLoS Comput. Biol..

[68] Mei S., Li F., Leier A., Marquez-Lago T.T., Giam K., Croft N.P., Akutsu T., Ian Smith A., Li J., Rossjohn J. (2020). A Comprehensive Review and Performance Evaluation of Bioinformatics Tools for HLA Class I Peptide-Binding Prediction. Brief. Bioinform..

[69] Koşaloğlu-Yalçın Z., Blazeska N., Carter H., Nielsen M., Cohen E., Kufe D., Conejo-Garcia J., Robbins P., Schoenberger S.P., Peters B. (2021). The Cancer Epitope Database and Analysis Resource: A Blueprint for the Establishment of a New Bioinformatics Resource for Use by the Cancer Immunology Community. Front. Immunol..

[70] Dimitrov I., Naneva L., Doytchinova I., Bangov I. (2014). AllergenFP: Allergenicity Prediction by Descriptor Fingerprints. Bioinformatics.

[71] Gupta S., Kapoor P., Chaudhary K., Gautam A., Kumar R., Raghava G.P.S. (2013). In Silico Approach for Predicting Toxicity of Peptides and Proteins. PLoS ONE.

[72] Meng X.-Y., Zhang H.-X., Mezei M., Cui M. (2011). Molecular Docking: A Powerful Approach for Structure-Based Drug Discovery. Curr. Comput. Aided Drug Des..

[73] Lee H., Heo L., Lee M.S., Seok C. (2015). GalaxyPepDock: A Protein–Peptide Docking Tool Based on Interaction Similarity and Energy Optimization. Nucleic Acids Res..

[74] Alam N., Schueler-Furman O. (2017). Modeling Peptide-Protein Structure and Binding Using Monte Carlo Sampling Approaches: Rosetta Flexpepdock and Flexpepbind. Methods Mol. Biol..

[75] London N., Raveh B., Cohen E., Fathi G., Schueler-Furman O. (2011). Rosetta FlexPepDock Web Server—High Resolution Modeling of Peptide–Protein Interactions. Nucleic Acids Res..

[76] Antes I. (2010). DynaDock: A New Molecular Dynamics-Based Algorithm for Protein–Peptide Docking Including Receptor Flexibility. Proteins Struct. Funct. Bioinform..

[77] Raveh B., London N., Zimmerman L., Schueler-Furman O. (2011). Rosetta FlexPepDock Ab-Initio: Simultaneous Folding, Docking and Refinement of Peptides onto Their Receptors. PLoS ONE.

[78] van Zundert G.C.P., Rodrigues J.P.G.L.M., Trellet M., Schmitz C., Kastritis P.L., Karaca E., Melquiond A.S.J., van Dijk M., de Vries S.J., Bonvin A.M.J.J. (2016). The HADDOCK2.2 Web Server: User-Friendly Integrative Modeling of Biomolecular Complexes. J. Mol. Biol..

[79] Rentzsch R., Renard B.Y. (2015). Docking Small Peptides Remains a Great Challenge: An Assessment Using AutoDock Vina. Brief. Bioinform..

[80] Yan C., Xu X., Zou X. (2016). Fully Blind Docking at the Atomic Level for Protein-Peptide Complex Structure Prediction. Structure.

[81] de Vries S.J., Rey J., Schindler C.E.M., Zacharias M., Tuffery P. (2017). The PepATTRACT Web Server for Blind, Large-Scale Peptide–Protein Docking. Nucleic Acids Res..

[82] Ben-Shimon A., Niv M.Y. (2015). AnchorDock: Blind and Flexible Anchor-Driven Peptide Docking. Structure.

[83] Kurcinski M., Jamroz M., Blaszczyk M., Kolinski A., Kmiecik S. (2015). CABS-Dock Web Server for the Flexible Docking of Peptides to Proteins without Prior Knowledge of the Binding Site. Nucleic Acids Res..

[84] Godfrey D.I., le Nours J., Andrews D.M., Uldrich A.P., Rossjohn J. (2018). Unconventional T Cell Targets for Cancer Immunotherapy. Immunity.

[85] Pfeiffer C., Stein J., Southwood S., Ketelaar H., Sette A., Bottomly K. (1995). Altered Peptide Ligands Can Control CD4 T Lymphocyte Differentiation in Vivo. J. Exp. Med..

[86] Scholz C., Hlllsberg P., Sette A., Hager D.A. (1995). Modulation of Cytokine Patterns of Human Autoreactive T Cell Clones by a Single Amino Acid Substitution of Their Peptide Ligand. Immunity.

[87] Robinson N.E., Robinson A.B. (2008). Use of Merrifield Solid Phase Peptide Synthesis in Investigations of Biological Deamidation of Peptides and Proteins. Biopolymers.

[88] Fabian H., Schultz C.P. (2000). Fourier Transform Infrared Spectroscopy in Peptide and Protein Analysis. Encyclopedia of Analytical Chemistry: Applications, Theory and Instrumentation.

[89] Siligardi G., Hussain R. (2015). CD Spectroscopy: An Essential Tool for Quality Control of Protein Folding. Methods Mol. Biol..

[90] Acikara O.B., Acikara O.B. (2013). Ion-Exchange Chromatography and Its Applications. Column. Chromatogr..

[91] Camperi S.A., Marani M., Camila M., Ceron M., Albericio F. (2010). Protein Purification by Affinity Chromatography with Peptide Ligands Selected from the Screening of Combinatorial Libraries. Trends Chromatogr..

[92] Calvo Tardón M., Allard M., Dutoit V., Dietrich P.Y., Walker P.R. (2019). Peptides as Cancer Vaccines. Curr. Opin. Pharm..

[93] Zhu S.Y., Yu K. (2022). da Breast Cancer Vaccines: Disappointing or Promising?. Front. Immunol..

[94] Fisk B., Blevins T.L., Wharton J.T., Ioannides C.G. (1995). Identification of an Immunodominant Peptide of HER-2/Neu Protooncogene Recognized by Ovarian Tumor-Specific Cytotoxic T Lymphocyte Lines. J. Exp. Med..

[95] Peoples G.E., Gurney J.M., Hueman M.T., Woll M.M., Ryan G.B., Storrer C.E., Fisher C., Shriver C.D., Ioannides C.G., Ponniah S. (2005). Clinical Trial Results of a HER2/Neu (E75) Vaccine to Prevent Recurrence in High-Risk Breast Cancer Patients. J. Clin. Oncol..

[96] Peoples G.E., Holmes J.P., Hueman M.T., Mittendorf E.A., Amin A., Khoo S., Dehqanzada Z.A., Gurney J.M., Woll M.M., Ryan G.B. (2008). Combined Clinical Trial Results of a HER2/Neu (E75) Vaccine for the Prevention of Recurrence in High-Risk Breast Cancer Patients: U.S. Military Cancer Institute Clinical Trials Group Study I-01 and I-02. Clin. Cancer Res..

[97] Mittendorf E.A., Clifton G.T., Holmes J.P., Schneble E., van Echo D., Ponniah S., Peoples G.E. (2014). Final Report of the Phase I/II Clinical Trial of the E75 (Nelipepimut-S) Vaccine with Booster Inoculations to Prevent Disease Recurrence in High-Risk Breast Cancer Patients. Ann. Oncol..

[98] Clifton G.T., Hale D., Vreeland T.J., Hickerson A.T., Litton J.K., Alatrash G., Murthy R.K., Qiao N., Philips A.V., Lukas J.J. (2020). Results of a Randomized Phase IIb Trial of Nelipepimut-S + Trastuzumab vs Trastuzumab to Prevent Recurrences in High-Risk HER2 Low-Expressing Breast Cancer Patients. Clin. Cancer Res..

[99] Carmichael M.G., Benavides L.C., Holmes J.P., Gates J.D., Mittendorf E.A., Ponniah S., Peoples G.E. (2010). Results of the First Phase 1 Clinical Trial of the HER-2/Neu Peptide (GP2) Vaccine in Disease-Free Breast Cancer Patients: United States Military Cancer Institute Clinical Trials Group Study I-04. Cancer.

[100] Mittendorf E.A., Ardavanis A., Litton J.K., Shumway N.M., Hale D.F., Murray J.L., Perez S.A., Ponniah S., Baxevanis C.N., Papamichail M. (2016). Primary Analysis of a Prospective, Randomized, Single-Blinded Phase II Trial Evaluating the HER2 Peptide GP2 Vaccine in Breast Cancer Patients to Prevent Recurrence. Oncotarget.

[101] Patel S.S., McWilliams D.B., Patel M.S., Fischette C.T., Thompson J., Daugherty F.J. (2021). Five Year Median Follow-up Data from a Prospective, Randomized, Placebo-Controlled, Single-Blinded, Multicenter, Phase IIb Study Evaluating the Reduction of Recurrences Using HER2/Neu Peptide GP2 + GM-CSF vs. GM-CSF Alone after Adjuvant Trastuzumab in HER2 Positive Women with Operable Breast Cancer. Cancer Res..

[102] Humphreys R.E., Adams S., Koldzic G., Nedelescu B., von Hofe E., Xu M. (2000). Increasing the Potency of MHC Class II-Presented Epitopes by Linkage to Ii-Key Peptide. Vaccine.

[103] Holmes J.P., Benavides L.C., Gates J.D., Carmichael M.G., Hueman M.T., Mittendorf E.A., Murray J.L., Amin A., Craig D., von Hofe E. (2008). Results of the First Phase I Clinical Trial of the Novel Ii-Key Hybrid Preventive HER-2/Neu Peptide (AE37) Vaccine. J. Clin. Oncol..

[104] Gates J.D., Clifton G.T., Benavides L.C., Sears A.K., Carmichael M.G., Hueman M.T., Holmes J.P., Jama Y.H., Mursal M., Zacharia A. (2010). Circulating Regulatory T Cells (CD4+CD25+FOXP3+) Decrease in Breast Cancer Patients after Vaccination with a Modified MHC Class II HER2/Neu (AE37) Peptide. Vaccine.

[105] Luo C., Wang P., He S., Zhu J., Shi Y., Wang J. (2022). Progress and Prospect of Immunotherapy for Triple-Negative Breast Cancer. Front. Oncol..

[106] Hijikata Y., Okazaki T., Tanaka Y., Murahashi M., Yamada Y., Yamada K., Takahashi A., Inoue H., Kishimoto J., Nakanishi Y. (2018). A Phase I Clinical Trial of RNF43 Peptide-Related Immune Cell Therapy Combined with Low-Dose Cyclophosphamide in Patients with Advanced Solid Tumors. PLoS ONE.

[107] Weber J.S., Kudchadkar R.R., Yu B., Gallenstein D., Horak C.E., Inzunza H.D., Zhao X., Martinez A.J., Wang W., Gibney G. (2013). Safety, Efficacy, and Biomarkers of Nivolumab with Vaccine in Ipilimumab-Refractory or -Naive Melanoma. J. Clin. Oncol..

[108] Naito M., Itoh K., Komatsu N., Yamashita Y., Shirakusa T., Yamada A., Moriya F., Ayatuka H., Mohamed E.R., Matsuoka K. (2008). Dexamethasone Did Not Suppress Immune Boosting by Personalized Peptide Vaccination for Advanced Prostate Cancer Patients. Prostate.

[109] Rettig L., Seidenberg S., Parvanova I., Samaras P., Knuth A., Pascolo S. (2011). Gemcitabine Depletes Regulatory T-Cells in Human and Mice and Enhances Triggering of Vaccine-Specific Cytotoxic T-Cells. Int. J. Cancer.

[110] Shirahama T., Muroya D., Matsueda S., Yamada A., Shichijo S., Naito M., Yamashita T., Sakamoto S., Okuda K., Itoh K. (2017). A Randomized Phase II Trial of Personalized Peptide Vaccine with Low Dose Cyclophosphamide in Biliary Tract Cancer. Cancer Sci..

[111] Kono K., Sato E., Naganuma H., Takahashi A., Mimura K., Nukui H., Fujii H. (2004). Trastuzumab (Herceptin) Enhances Class I-Restricted Antigen Presentation Recognized by HER-2/Neu-Specific T Cytotoxic Lymphocytes. Clin. Cancer Res..

[112] Petricevic B., Laengle J., Singer J., Sachet M., Fazekas J., Steger G., Bartsch R., Jensen-Jarolim E., Bergmann M. (2013). Trastuzumab Mediates Antibody-Dependent Cell-Mediated Cytotoxicity and Phagocytosis to the Same Extent in Both Adjuvant and Metastatic HER2/Neu Breast Cancer Patients. J. Transl. Med..

[113] Gall V.A., Philips A.V., Qiao N., Clise-Dwyer K., Perakis A.A., Zhang M., Clifton G.T., Sukhumalchandra P., Ma Q., Reddy S.M. (2017). Trastuzumab Increases HER2 Uptake and Cross-Presentation by Dendritic Cells. Cancer Res..

[114] Hickerson A., Clifton G.T., Hale D.F., Peace K.M., Holmes J.P., Vreeland T.J., Litton J.K., Murthy R.K., Lukas J.J., Mittendorf E.A. (2019). Final Analysis of Nelipepimut-S plus GM-CSF with Trastuzumab versus Trastuzumab Alone to Prevent Recurrences in High-Risk, HER2 Low-Expressing Breast Cancer: A Prospective, Randomized, Blinded, Multicenter Phase IIb Trial. J. Clin. Oncol..

